# Regional disparity in epidemiological characteristics of adolescent scoliosis in China: Data from a screening program

**DOI:** 10.3389/fpubh.2022.935040

**Published:** 2022-12-06

**Authors:** Jiaoling Huang, Xuan Zhou, Xin Li, Haibin Guo, Yuqi Yang, I. O. Hong Cheong, Qing Du, Hui Wang

**Affiliations:** ^1^School of Public Health, Shanghai Jiao Tong University School of Medicine, Shanghai, China; ^2^Department of Rehabilitation Medicine, Xinhua Hospital Affiliated to the Shanghai Jiao Tong University School of Medicine, Shanghai, China; ^3^College of Global Public Health, New York University, New York, NY, United States

**Keywords:** scoliosis, regional disparity, school scoliosis screening, China, adolescence

## Abstract

**Objective:**

We investigated regional disparities in rates of scoliosis among adolescents in western and eastern China and the dominant factors underlying these disparities.

**Methods:**

This cross-sectional study used data from a school scoliosis screening program conducted in two typical areas: Yangpu District of Shanghai (eastern China) and Tianzhu Tibetan Autonomous County of Gansu Province (western China), during October 2020 to February 2021. Participants included adolescents aged 12–16 years (4,240 in Shanghai and 2,510 in Gansu Province). School scoliosis screening data were obtained on age, sex, height, weight and BMI, and region as well. We screened angles of trunk rotation in level of proximal thoracic (T1–T4), main thoracic (T5–T12), and lumbar (T12–L4) by the forward bend test with scoliometer. An angle of trunk rotation ≥5° was used as the criterion to identify suspected scoliosis.

**Results:**

The proportion of suspected scoliosis was lower in Shanghai (6.9%) than in Gansu (8.6%). Angle of trunk rotation tended to increase with age in Shanghai, peaking at 15 years, but decreased with age in Gansu, and bottomed at 15 years. The angle of trunk rotation in the proximal thoracic, main thoracic, and lumbar part of the spine appeared to be larger in Gansu adolescents and in Shanghai female adolescents. Age was a relevant factor in angle trunk rotation in regression models and interacted with region as well.

**Conclusion:**

We found regional and age- and sex-related disparities in rates of suspected scoliosis.

## Introduction

Scoliosis is a common condition in adolescents, with a reported prevalence of 2–3% in the literature globally (1, 2), and higher prevalence of 8.3–9.3% were reported using X-ray screening method (3, 4). Adolescent scoliosis develops at the age of 11–18 years and accounts for most cases (5). Unlike obesity and myopia, which are easier to identify in schoolchildren and have a higher prevalence, scoliosis has received little attention (6). However, scoliosis can progress rapidly and develop into a disability if it is not diagnosed and treated promptly (7–10).

School screening is an important measure of identifying scoliosis (11), and is mandated in some parts of the United States (12). China published *National Monitoring Program for Common Diseases and Health Influencing Factors of Students* in 2018 and selected schools and students for fixed-point monitoring of common conditions since then, including scoliosis (13). The prevalence of scoliosis among adolescents in China is 0.11–2.64% (14), and the monitoring takes place mostly in the eastern and southern regions of the country, which are distributed in coastal areas and more developed (15). Western China differs from these regions in geography, climate, culture, demographic characteristics, and social economy, especially in areas where ethnic minorities gather, and these disparities may affect the prevalence and outcomes of scoliosis in adolescents.

Data on regional disparities in adolescent scoliosis in China are scarce. In the southeastern Guangdong Province, a study of 99,695 students aged 10–19 years estimated the prevalence of idiopathic scoliosis at 5.14% (16). An older study from the 1980s estimated the prevalence in Shanghai at 15% (17). A systematic review and meta-analysis concluded that rates were relatively low in eastern and southern China, and decreased with latitude (14). However, epidemiological information on scoliosis in the western region of the country is almost non-existent.

In the present study, we selected two typical areas in China, representing the west and the east, to compare scoliosis rates and disparities in adolescents and investigate the factors involved.

## Materials and methods

This study was by the Ethics Committee of Xinhua Hospital affiliated to the Shanghai Jiao Tong University School of Medicine. A parent or guardian assented to student participation before they completed the survey. This study is reported following the Strengthening the Reporting of Observational Studies in Epidemiology (STROBE) reporting guideline for cross-sectional studies.

### Study design and participants

The school scoliosis screening program was conducted in Yangpu District of Shanghai and in the Tianzhu Tibetan Autonomous County in Gansu Province. Yangpu District, one of the city central areas, is a representative urban area in Shanghai, while the Tianzhu Tibetan Autonomous County, a typical multi-ethnic settlement with a recent history of poverty, is a typical area in Gansu Province. We screened adolescents aged 12–16 years. The minimum required sample was calculated as 3,457 in Shanghai and 1,580 in Gansu (5). To allow for losses to follow-up, we screened 4,240 students at 10 junior high schools in Shanghai (30% of junior high school students in Shanghai public schools) and 2,510 students at two junior high schools in Gansu (50% of junior high school students in Tianzhu). Written informed consent was obtained from parents of all participants.

### Procedures

The screening form was designed to collect basic information and the angle of trunk rotation (ATR) at three levels: proximal thoracic (T1–T4), main thoracic (T5–T12), and lumbar (T12–L4) (18). The screening forms were sent to schools in advance and students and their parents were asked to fill in basic information. Two screening teams, each composed of two examiners, a data recorder, and a supervisor, were sent to each school. The screening was conducted in well-lit rooms with moderate temperature and segregated by sex. Students wore thin and tight clothes, with long hair pulled up to provide full view of the back. The examination began with the students standing straight and facing away from the examiner, head up and the arms relaxed at the sides. The examiner used a scoliometer to measure the ATR in the Adam's forward test position. The students bent their trunk forward until it was perpendicular to the ground, keeping the palms of their hands together. All data were input into EpiData on the day of the screening, and the supervisor randomly selected 10% of entered data to compare with the original form data for quality control.

### Scoliosis screening criterion

The forward bend test is the most common screening technique used globally to measure ATR. Frequency analysis has shown its relatively good specificity, sensitivity, and predictive ability (19, 20). The correlation between a scoliometer measurement and a radiographic measurement has been calculated as 0.7 (*p* < 0.05) (21). In this study, an ATR ≥5° was used as the criterion for positive screening, as suggested by SOSORT guidelines (19). In students whose ATR was determined as ≥5° in the first measurement, the test was repeated.

### Statistical analysis

All statistical analyses were conducted in SPSS 25.0 and GraphPad Prism 8.0. Descriptive analyses were used to capture the sample characteristics, and the Mann-Whitney *U*-test (A non-parametric test for this non-normally distributed sample) and Pearson χ^2^ test were performed to analyze between-group differences in continuous and categorical variables. Two-factor analysis of variance was used to analyze the interaction between variables. Linear regression models were constructed to analyze the risk factors for scoliosis. Normal distribution of residuals were analyzed in advance which were normally distributed, satisfying the premise of linear regression. All variables, including significant interaction variables, were incorporated into the models after excluding collinearity. Propensity score matching (PSM) was used to fit two samples matched. *P*-values were two-sided, and statistical significance was set at *p* < 0.05.

And before regression model performed, we analyzed the normal distribution of residuals, and it is normally distributed, satisfying the premise of linear regression.

## Results

Table 1 lists the main characteristics and trunk asymmetry values in the participants. Study participants were significantly younger in Shanghai (13.16 ± 1.13 years) than in Gansu (14.11 ± 0.96 years), as Shanghai recruits preparatory class students (the sixth grade) in junior high schools. Male students accounted for 51.18 and 50.80% of participants in Shanghai and Gansu, respectively. Adolescents in Gansu were taller but weighed less, yielding a lower body mass index (BMI). ATR was higher in the Gansu participants at level of proximal thoracic and main thoracic but lower at lumbar. The maximum ATR recorded was 2.73 ± 1.44° in Shanghai and 2.67 ± 1.44° in Gansu. At all measurement levels, the positive rate of screening was higher in Gansu (8.6%) than in Shanghai (6.9%). After PSM was conducted, the regional disparities were still statistically significant in lumbar and max level (Supplementary Table 1). Supplementary Tables 2, 3 contains more detailed information on ATR distribution in two regions without and with PSM.

**Table 1:** Characteristics of the study population.

	**Shanghai** **(*N* = 4,240)**	**Gansu** **(*N* = 2,510)**	***p*** **-value**
**Age, years**	13.16 (1.13)	14.11 (0.96)	< 0.001^**^
~12 year	1485 (35.0)	80 (3.2)	< 0.001^**^
13 year	1233 (29.1)	621 (24.7)	
14 year	896 (21.1)	930 (37.1)	
15 year	586 (13.8)	734 (29.2)	
16 year~	40 (0.9)	145 (5.8)	
Sex, Male (%)	2179 (51.18)	1275 (50.80)	0.761
Weight, kg	54.57 (13.91)	50.99 (9.62)	< 0.001^**^
Height, cm	162.06 (8.84)	162.71 (8.51)	0.009^**^
**BMI, kg/m** ^**2**^	20.59 (4.10)	19.22 (3.14)	< 0.001^**^
~12 year	19.82 (3.81)	19.00 (2.74)	0.108
13 year	20.36 (4.12)	18.92 (3.54)	< 0.001^**^
14 year	21.30 (4.20)	19.23 (3.20)	< 0.001^**^
15 year	21.81 (4.15)	19.45 (2.78)	< 0.001^**^
16 year~	22.55 (3.77)	19.36 (2.85)	< 0.001^**^
**ATR (°)**			
**Proximal thoracic**	1.47 (1.03)	1.57 (1.04)	0.003^**^
ATR≥5°	26 (0.6)	35 (1.4)	0.001^**^
**Main thoracic**	1.93 (1.38)	2.03 (1.37)	0.007^**^
ATR≥5°	176 (4.2)	122 (4.9)	0.170
**Lumbar**	2.09 (1.51)	2.01 (1.43)	0.001^**^
ATR≥5°	188 (4.4)	131 (5.2)	0.142
**Max**	2.73 (1.44)	2.67 (1.44)	0.007^**^
ATR≥5°	292 (6.9)	216 (8.6)	0.010^**^

ATR, angle of trunk rotation; BMI, body mass index; Proximal thoracic (T1–T4); main thoracic (T5–T12); lumbar (T12–L4); Max, maximal value observed. Data are *n* (%) or mean (SD).

** *p* < 0.01.

**Figure 1:**
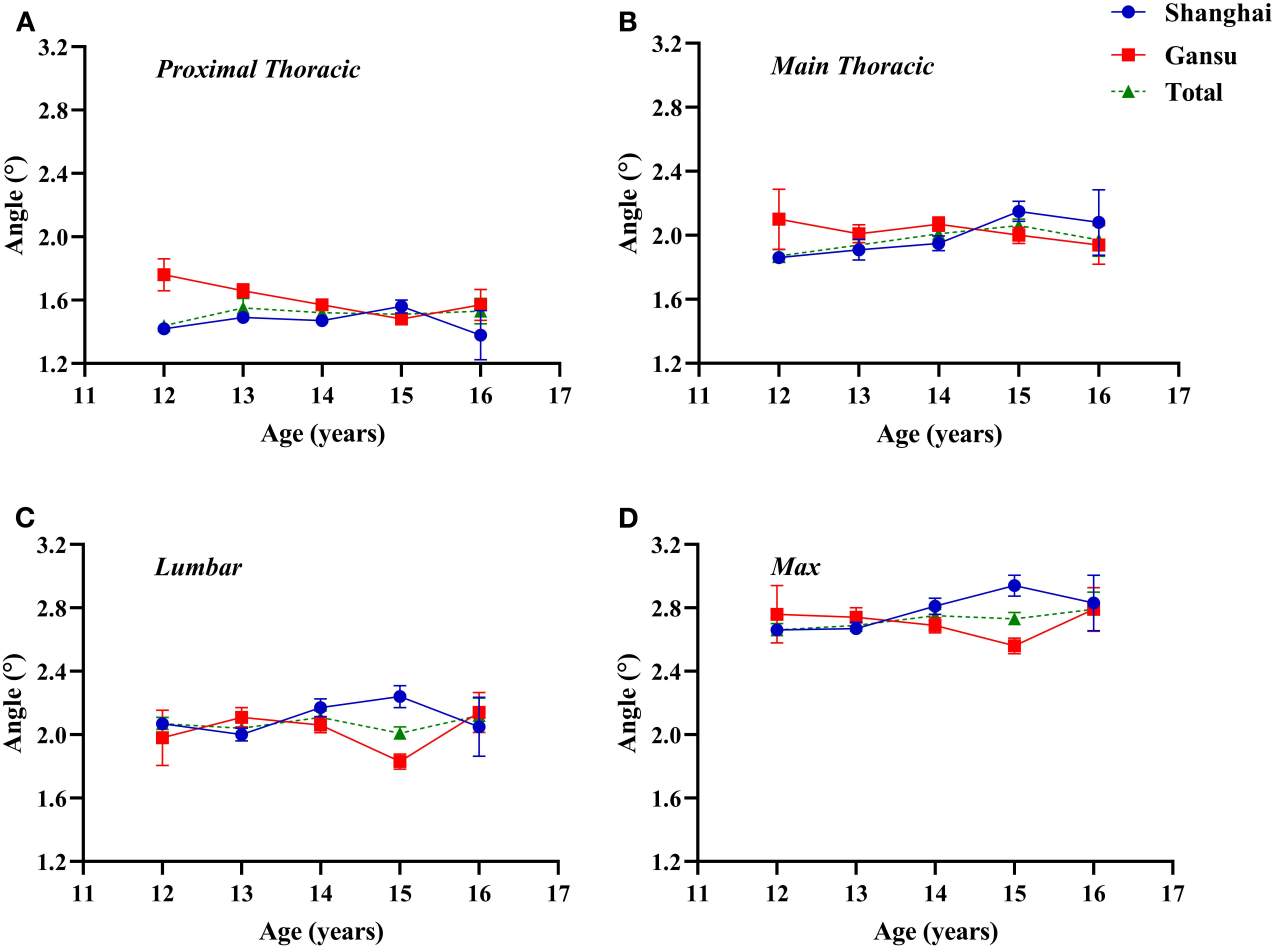
Two-factor analysis of variance to assess differences in angles of trunk rotation by region and age. **(A)** Proximal thoracic (T1–T4); **(B)** Main thoracic (T5–T12); **(C)** Lumbar (T12–L4); **(D)** Max, maximal value observed.

In Shanghai, ATR tended to increase with age and peaked in 15-year-old students, whereas in Gansu, ATR tended to decrease with age (Figure 1). Specifically, adolescents in Gansu had higher ATR values at level of proximal thoracic (*F* = 9.346, *p* = 0.002), especially among students aged 12 and 13 years (12 years: Z = −3.208, *p* = 0.001; 13 years: Z = −2.736, *p* = 0.006), but this was reversed among 15-year-old students (Z = −2.278, *p* = 0.023). At the other two levels, there were no significant differences between the two regions. Interaction effect of age and region was statistically significant (age × region, proximal thoracic: *F* = 4.301, *p* = 0.003; main thoracic: *F* = 2.649, *p* = 0.032; lumbar: *F* = 5.515, *p* < 0.0001; maximum: *F* = 4.994, *p* = 0.001).

**Figure 2:**
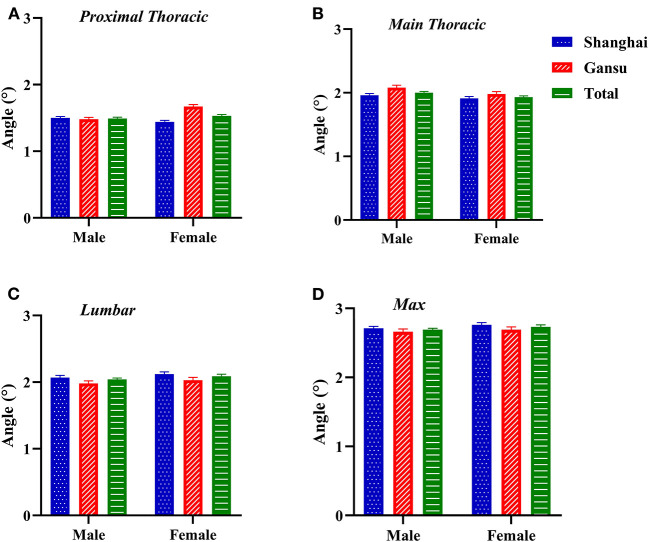
Two-factor analysis of variance to assess differences in angles of trunk rotation by region and sex. **(A)** Proximal thoracic (T1–T4); **(B)** Main thoracic (T5–T12); **(C)** Lumbar (T12–L4); **(D)** Max, maximal value observed.

Proximal thoracic ATR was higher in female students, especially in Gansu (total ATR: 1.51 ± 1.04°, Z = −1.977, *p* = 0.048; Gansu ATR: 1.57 ± 1.04°, Z = −5.731, *p* < 0.0001; Figure 2). Similarly, lumbar and maximal ATRs were highest in female students in Gansu (lumbar ATR: 2.03 ± 1.48°, Z = −0.661, *p* = 0.509; maximal ATR: 2.69 ± 1.43, Z = −1.157, *p* = 0.247). Main thoracic ATRs were highest in male students in Gansu (2.08 ± 1.38°, Z = −1.683, *p* = 0.092). Interaction of region and sex was not significant except at proximal thoracic (proximal thoracic: *F* = 23.119, *p* < 0.0001; main thoracic: *F* = 0.431, *p* = 0.512; lumbar: *F* = 0.007, *p* = 0.935; maximal values: *F* = 0.132, *p* = 0.717).

**Figure 3:**
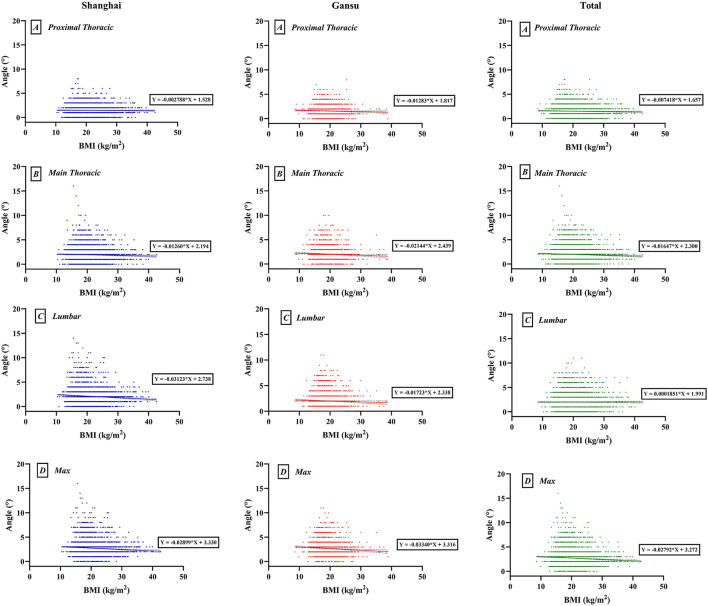
Scatter map of interaction between body mass index (BMI) and angles of trunk rotation. **(A)** Proximal thoracic (T1–T4); **(B)** Main thoracic (T5–T12); **(C)** Lumbar (T12–L4); **(D)** Max, maximal value observed.

We found a weak negative correlation between BMI and ATR, which varied by region (maximal: Shanghai, *r* = −0.083, *p* < 0.0001; Gansu, *r* = −0.073, *p* < 0.0001; total, *r* = −0.074, *p* < 0.0001; Figure 3). At level of proximal thoracic, the overall negative correlation was significant, but in Shanghai or Gansu alone, there was no obvious correlation (proximal thoracic: Shanghai, *r* = −0.011, *p* = 0.470; Gansu, *r* = −0.039, *p* = 0.053; total, *r* = −0.027, *p* = 0.024). At level of main thoracic, there was a negative correlation in both Shanghai and Gansu (Shanghai, *r* = −0.037, *p* = 0.015; Gansu, *r* = −0.049, *p* = 0.014; total, *r* = −0.046, *p* < 0.0001). At level of lumbar, only Shanghai had a negative correlation between BMI and ATR (Shanghai, *r* = −0.085, *p* < 0.0001; Gansu, *r* = −0.038, *p* = 0.058; total, *r* = −0.064, *p* < 0.0001).

Linear regression models were constructed separately by level and region (Supplementary Table 4). Female students had a larger ATR than male students at all levels and were more likely to be diagnosed with scoliosis (maximal values: Shanghai, S.β = 0.020, *p* = 0.216; Gansu, S.β = 0.048, *p* = 0.027). ATR decreased as BMI decreased (maximal values: Shanghai, S.β = −0.110, *p* < 0.0001; Gansu: S.β = −0.068, *p* = 0.0001). Age differences were notable between the two regions. In Shanghai, ATR increased with age (proximal thoracic: β = 0.019, *p* = 0.297; main thoracic: β = 0.043, *p* = 0.018; lumbar: β = 0.030, *p* = 0.102; maximal: S.β = 0.052, *p* = 0.004). In Gansu, ATR decreased with age (proximal thoracic: β = −0.081, *p* < 0.0001; main thoracic: β = −0.050, *p* = 0.018; lumbar: β = −0.069, *p* = 0.001; maximal: S.β = −0.064, *p* = 0.003).

We then added the interaction of region and age to the linear regression models, and the results indicated that ATR changes with age varied by region at all measurement levels (Table 2). There was a significant interaction between age and region (model 1: S.β = −0.066, *p* < 0.0001; model 2: S.β = −0.045, *p* = 0.006; model 3: S.β = −0.057, *p* < 0.0001; model 4: S.β = −0.190, *p* = 0.029). The linear regression model results after PSM (Supplementary Table 5) were similar to the results in Table 2.

**Table 2:** Multivariate linear regression models of age, region, and the interaction of age and region.

**Independent variable**	**Unstandardized coefficient**		**Standardized coefficient**	**95% CI for** ***β***		**t**	***P*** **-value**	**Collinearity statistics**
	**β**	**S. E**	**S. β**	**Lower**	**Upper**			**Tolerance**	**VIF**
*Model 1 (Proximal thoracic)*									
Region (reference = Shanghai)	0.135	0.030	0.063	0.075	0.194	4.447	< 0.0001**	0.739	1.353
Age_c	0.015	0.016	0.017	−0.016	0.046	0.939	0.348	0.470	2.130
Region × Age_c	−0.106	0.026	−0.066	−0.156	−0.055	−4.073	< 0.0001**	0.557	1.794
*Model 2 (Main thoracic)*									
Region (reference = Shanghai)	0.075	0.040	0.026	−0.004	0.153	1.867	0.062	0.739	1.353
Age_c	0.039	0.021	0.033	−0.002	0.080	1.859	0.063	0.470	2.130
Region × Age_c	−0.095	0.034	−0.045	−0.162	−0.027	−2.757	0.006**	0.557	1.794
*Model 3 (Lumbar)*									
Region (reference = Shanghai)	−0.094	0.043	−0.031	−0.179	−0.009	−2.181	0.029*	0.739	1.353
Age_c	0.034	0.023	0.027	−0.010	0.079	1.530	0.126	0.470	2.130
Region × Age_c	−0.130	0.037	−0.057	−0.202	−0.057	−3.515	< 0.0001**	0.557	1.794
*Model 4 (Max)*									
Region (reference = Shanghai)	−0.088	0.042	−0.03	−0.17	−0.007	−2.121	0.034*	0.739	1.353
Age_c	0.057	0.022	0.046	0.015	0.1	2.635	0.008**	0.470	2.130
Region × Age_c	−17.65	7.870	−0.190	−33.42	−1.89	−2.243	0.029*	0.957	1.045

The models include body mass index and sex; those data were excluded from this table for simplicity. Age data were centralized to prevent collinearity after the interaction of the two variables. Proximal thoracic (T1−T4); main thoracic (T5−T12); lumbar (T12−L4); Max, maximal value observed.

CI, confidence interval; VIF, variance inflation factor.

The * symbol indicates the value of *p* < 0.05 and the ** symbol indicates the value of *p* < 0.01.

## Discussion

ATRs exceeding 5° are considered an indication of scoliosis and referred to whole-spine radiographs (22, 23). We found a higher proportion of suspected scoliosis in Gansu, consistent with a higher prevalence (24). The prevalence of scoliosis in China varies by region from 0.11 to 2.52% (25–28). However, adolescent idiopathic scoliosis has no clear cause, and is generally considered to be multifactorial (29–31). A study of 1.2 million students in Japan reported a higher incidence in densely populated areas than in sparsely populated districts (32).

In Shanghai, ATR increased with age and peaked at 15, then decreased. Such an upward trend in the prevalence of scoliosis has been observed elsewhere (33) and may be related to the popularization of screening and to increased schoolwork pressure (34, 35). However, in Gansu, ATR decreased with age and bottomed at 15, then increased. This contrast is uncommon, and may correlate with allocation of education resources, which are relatively scarce in western China (36). A cornerstone policy to speed up the popularization of high school education in the western area was implemented in 2016 (37). In 2019, the State Council proposed to implement the Central and Western Education Revitalization and Development Plan, which covered the Tianzhu Tibetan Autonomous County (38). The learning burdens of lower-grade students are heavier with the implementation of various education policies (39), and that may promote the development of scoliosis (40).

Another interesting finding was sex disparities between Shanghai and Gansu. ATR among female students was higher in Gansu at level of proximal thoracic, and may be related to the higher fertility rate in western China, as the one-child policy varies between regions (41, 42). The one-child policy does not apply in the Tianzhu autonomous region, and female adolescents typically care for younger siblings and often hold or carry them. Specific asymmetrical exercises are known to increase electromyography amplitudes of the paraspinal muscles in the concavity, which may promote scoliosis in the proximal thoracic (43). ATRs in the main thoracic were higher in male students, especially in Gansu, which might be correlated with regional development and family role of male adolescents in families. Male students in Gansu often undertake some manual labor such as carrying heavy objects which is proved to be negatively correlated with spine health, especially for the main thoracic, while counterparts in Shanghai are not requested to contribute to the family in their early age (44, 45). Surprisingly, ATRs of the lumbar part appeared to be larger in Shanghai, especially in female students. It is widely accepted that the study burden and pressure in Shanghai is greater than in other cities in China, and female adolescents are more likely to be sedentary rather than physically active (46, 47). Studies have provided evidence that there was a positive relationship between long sedentary time and scoliosis, and negative relationship between enough physical activity time and scoliosis (48–50).

We found that ATR was negatively correlated with BMI both in Shanghai and Gansu, i.e., a lower BMI was associated with a larger ATR. Slight differences in correlation coefficients were observed for the Gansu and Shanghai datasets. In terms of the main thoracic part, the absolute correlation coefficient was larger in Gansu than that in Shanghai, while the coefficient for the lumbar area was larger in Shanghai. Similar findings were observed in other studies, although the explanations contrast (51). One explanation was that obesity might affect the accurate measurement of ATR, and thus that scoliosis is difficult to identify in obese individuals (52). However, scoliosis may be less likely in obese adolescents because of abnormal leptin bioavailability (53). Nonetheless, overweight patients with adolescent idiopathic scoliosis have been reported to have a larger curve magnitude and be at higher risk of post-operative complications (54).

In linear regression models, we found that sex, age, and BMI were all associated with ATR, and age was the most notable factor. An interaction of region and age was also significant, and may perhaps be explained by education policy.

### Strength and limitation

This is the first study on region disparity of scoliosis in China. There are some limitations in this study. First, we compared two typical areas in western and eastern China. Firstly, it seems not enough to investigate age, gender and BMI as covariates, and more demographic and socioeconomic factors could be considered. Secondly, we did not perform X-ray examinations in this program considering medical expense and healthcare resource, which might decrease the accuracy of scoliosis measurements, as less developed areas in China may lack adequate healthcare funding and resources to support X-ray screening. Thirdly, according to current studies, adolescents aged from 10 to 14 were more prone to develop scoliosis, who should be paid special attention. However, this study only screened junior school students as research object aging from 12 to 16, thus students in primary school aging from 10 to 11 were missed. Lastly, it was a pity that our research did not collect follow-up diagnosis data, due to the inconvenience caused by the COVID-19 and the long distance.

## Conclusion

We found a smaller proportion of suspected scoliosis in Shanghai than Gansu but a higher proportion of ATR exceeding 5°, and ATR also varied by age and sex.

## Data availability statement

Owing to the private nature of spine health among adolescents in this study, the data are not publicly available but may be obtained from the corresponding author on reasonable academic request.

## Ethics statement

The studies involving human participants were reviewed and approved by the Ethics Committee of Xin Hua Hospital affiliated to the Shanghai Jiao Tong University School of Medicine (XHEX-D-2021-073). The patients/participants provided their written informed consent to participate in this study. Written informed consent was obtained from the individual(s) for the publication of any potentially identifiable images or data included in this article.

## Author contributions

JH and XL conducted the literature review and drafted the outline of the manuscript. HG and YY contributed to the analysis and interpretation. XZ contributed to the overall design of the paper, led the analysis, and interpretation of data. IC contributed to the discussion and overall paper. QD and HW did critical revisions, approved the final version for publication, and agreed to be accountable for all aspects of the work in ensuring that questions related to the accuracy or integrity of any part of the work are appropriately investigated and resolved. All authors contributed to the article and approved the submitted version.

## Funding

It was funded by National Natural Science Foundation of China (81972030 and 82030099) and the National Key R&D Program of China (2018YFC2000700).

## Conflict of interest

The authors declare that the research was conducted in the absence of any commercial or financial relationships that could be construed as a potential conflict of interest.

## Publisher's note

All claims expressed in this article are solely those of the authors and do not necessarily represent those of their affiliated organizations, or those of the publisher, the editors and the reviewers. Any product that may be evaluated in this article, or claim that may be made by its manufacturer, is not guaranteed or endorsed by the publisher.
