# Supplementary material for: Regional disparity in epidemiological characteristics of adolescent scoliosis in China: Data from a screening program
Source: Front Public Health. 2022 Dec 6;10:935040. doi: 10.3389/fpubh.2022.935040 (PMC9764629; doi:10.3389/fpubh.2022.935040)
Supplement: Supplementary file 1 [file Table_1.docx]

**eTable 1: Characteristics of the study population (PSM)**

|  | **Shanghai(N=1903)** | | **Gansu(N=1903)** | ***p*-Value** |
| --- | --- | --- | --- | --- |
| **Age, years** | 13.81(1.05) | 13.86(0.86) | | 0.659 |
| **Sex, Male (%)** | 893(46.9) | 950(49.9) | | 0.064 |
| **Weight, kg** | 51.85(10.49) | 51.64(10.07) | | 0.589 |
| **Height, cm** | 162.54(8.36) | 162.51(8.43) | | 0.402 |
| **BMI, kg/m^2^** | 19.55(3.33) | 19.48(3.17) | | 0.466 |
| **ATR (°)** |  |  | |  |
| **Proximal Thoracic** | 1.53(1.06) | 1.58(1.04) | | 0.382 |
| **ATR≥5°** | 17(0.9) | 27(1.4) | | 0.129 |
| **Main Thoracic** | 2.05(1.49) | 2.03(1.39) | | 0.862 |
| **ATR≥5°** | 95(5.0) | 100(5.3) | | 0.713 |
| **Lumbar** | 2.19(1.62) | 2.03(1.45) | | <0.001** |
| **ATR≥5°** | 106(5.6) | 111(5.8) | | 0.727 |
| **Max** | 2.87(1.57) | 2.68(1.45) | | <0.001** |
| **ATR≥5°** | 164(8.7) | 178(9.4) | | 0.427 |

**Note: PSM, propensity score matching; ATR, angle of trunk rotation; BMI, body mass index. Data are n (%) or mean (SD). ***p* < 0.01.**
