# Supplementary material for: Regional disparity in epidemiological characteristics of adolescent scoliosis in China: Data from a screening program
Source: Front Public Health. 2022 Dec 6;10:935040. doi: 10.3389/fpubh.2022.935040 (PMC9764629; doi:10.3389/fpubh.2022.935040)
Supplement: Supplementary file 2 [file Table_2.docx]

**eTable 2: Demographics of the study population by three standards.**

|  | **Region** | **Chinese standard**^1^ | | | | | **International standard 1**^2^ | | | | **International standard 2**^3^ | | |
| --- | --- | --- | --- | --- | --- | --- | --- | --- | --- | --- | --- | --- | --- |
| **ATR** |  | **[0,5)** | **[5,7)** | **[7,10)** | **10~** | **Chi-square** | **[0,4)** | **[4,7)** | **7~** | **Chi-square** | **[0,5)** | **5~** | **Chi-square** |
| **Proximal Thoracic** | **Shanghai, *N(%)*** | 4214(99.4) | 22(0.5) | 4(0.1) | - | 12.39** | 4068(95.9) | 168(4.0) | 4(0.1) | 3.40 | 4214(99.4) | 26(0.6) | 10.75* |
|  | **Gansu, *N(%)*** | 2475(98.6) | 33(1.3) | 2(0.1) | - |  | 2385(95.0) | 123(4.9) | 2(0.1) |  | 2475(98.6) | 35(1.4) |  |
|  | **Shanghai**  ***VS* Gansu, *Mean(SD)*** | 1.44(0.98)  1.52(0.94) * | 5.45(0.51)  5.24(0.44) | 7.5(0.58)  7.50(0.71) | - |  | 1.35(0.86)  1.42(0.83)* | 4.19(0.52)  4.33(0.60)** | 7.50(0.58)  7.50(0.71) |  | 1.44(0.98)  1.52(0.94)* | 5.77(0.91)  5.37(0.69)* |  |
| **Main Thoracic** | **Shanghai, *N(%)*** | 4064(95.8) | 125(2.9) | 45(1.1) | 6(0.1) | 2.67 | 3810(89.9) | 379(8.9) | 51(1.2) | 4.34 | 4064(95.8) | 176(4.2) | 1.88 |
|  | **Gansu, *N(%)*** | 2388(95.1) | 92(3.7) | 27(1.1) | 3(0.1) |  | 2217(88.3) | 263(10.5) | 30(1.2) |  | 2388(95.1) | 122(4.9) |  |
|  | **Shanghai**  ***VS* Gansu, *Mean(SD)*** | 1.76(1.05)  1.83(1.04)* | 5.36(0.48) 5.45(0.50) | 7.24(0.53)  7.19(0.40) | 12.00(2.53)  10.0(0.00) |  | 1.61(0.91)  1.66(0.88) | 4.45(0.70)  4.51(0.56) | 7.80(1.81)  7.47(0.94) |  | 1.76(1.05)  1.83(1.04)* | 6.07(1.53)  5.94(1.08) |  |
| **Lumbar** | **Shanghai, *N(%)*** | 4052(95.6) | 107(2.5) | 66(1.6) | 15(0.4) | 11.35** | 3725(87.9) | 434(10.2) | 81(1.9) | 0.92 | 4052(95.6) | 188(4.4) | 2.16 |
|  | **Gansu, *N(%)*** | 2379(94.8) | 91(3.6) | 38(1.5) | 2(0.1) |  | 2215(88.2) | 255(10.2) | 40(1.6) |  | 2379(94.8) | 131(5.2) |  |
|  | **Shanghai**  ***VS* Gansu, *Mean(SD)*** | 1.88(1.10)  1.78(1.01)** | 5.46(0.50)  5.47(0.50) | 7.59(0.74)  7.39(0.60) | 11.13(1.30)  11.00(0.00) |  | 1.70(0.95)  1.62(0.89)* | 4.36(0.68^j^  4.53(0.77)** | 8.25(1.63)  7.58(0.98) |  | 1.88(1.10)  1.78(1.05)** | 6.66(1.79)  6.11(1.19)* |  |
| **Max** | **Shanghai, *N(%)*** | 3948(93.1) | 174(4.1) | 99(2.3) | 19(0.4) | 16.55** | 3401(80.2) | 721(17.0) | 118(2.8) | 1.18 | 3948(93.1) | 292(6.9) | 6.69** |
|  | **Gansu, *N(%)*** | 2294(91.4) | 151(6.0) | 61(2.4) | 4(0.2) |  | 1994(79.4) | 451(18.0) | 65(2.6) |  | 2294(91.4) | 216(8.6) |  |
|  | **Shanghai**  ***VS* Gansu, *Mean(SD)*** | 2.45(0.93)  2.35(0.96)** | 5.49(0.50)  5.50(0.50) | 7.44(0.67)  7.31(0.53) | 11.26(1.70)  10.50(0.58) |  | 2.20(0.74)  2.10(0.76)** | 4.36(0.68)  4.50(0.77)** | 8.06(1.68)  7.51(0.94)* |  | 2.45(0.93)  2.35(0.96)** | 6.53(1.70)  6.11(1.13)* |  |

**Note: Data are n (%) or mean (SD) unless specified otherwise. ATR=the angle of trunk rotation. * *p*＜0.05, ** *p*＜0.01.**

**Reference**

1. GB/T 16133—2014. Screening of spinal curvature abnormality of children and adolescents[S]. In. People's Republic of China: National Health and Family Planning Commission of the People's Republic of China, China National Standardization Management Committee; 2015.

2. Adamczewska K, Wiernicka M, Malchrowicz-Mośko E, Małecka J, Lewandowski J. The Angle of Trunk Rotation in School Children: A Study from an Idiopathic Scoliosis Screening. Prevalence and Optimal Age Screening Value. *Int J Environ Res Public Health.* 2019;16(18):3426.

3. Negrini S, Donzelli S, Aulisa AG, et al. 2016 SOSORT guidelines: orthopaedic and rehabilitation treatment of idiopathic scoliosis during growth. *Scoliosis Spinal Disord.* 2018;13:3-3.
