# Supplementary material for: Regional disparity in epidemiological characteristics of adolescent scoliosis in China: Data from a screening program
Source: Front Public Health. 2022 Dec 6;10:935040. doi: 10.3389/fpubh.2022.935040 (PMC9764629; doi:10.3389/fpubh.2022.935040)
Supplement: Supplementary file 3 [file Table_3.docx]

**eTable 3: Demographics of the study population by three standards (PSM).**

|  | **Region** | **Chinese standard**^1^ | | | | | **International standard 1**^2^ | | | | **International standard 2**^3^ | | | |
| --- | --- | --- | --- | --- | --- | --- | --- | --- | --- | --- | --- | --- | --- | --- |
| **ATR** |  | **[0,5)** | **[5,7)** | **[7,10)** | **10~** | **Chi-square** | **[0,4)** | **[4,7)** | **7~** | **Chi-square** | **[0,5)** | **5~** | **Chi-square** |  |
| **Proximal Thoracic** | **Shanghai, *N(%)*** | 1886(99.1) | 14(0.7) | 3(0.2) | - | 4.63 | 1810(95.1) | 90(4.7) | 3(0.2) | 1.09 | 1886(99.1) | 17(0.9) | 2.30 |  |
|  | **Gansu, *N(%)*** | 1876(98.6) | 26(1.4) | 1(0.1) | - |  | 1808(95.0) | 94(4.9) | 1(0.1) |  | 1876(98.6) | 27(1.4) |  |  |
|  | **Shanghai**  ***VS* Gansu, *Mean(SD)*** | 1.50(0.99)  1.52(0.94) | 5.36(0.50)  5.23(0.43) | 7.33(0.58)  7.00(/) | - |  | 1.39(0.86)  1.43(0.83) | 4.21(0.53)  4.34(0.60) | 7.33(0.58)  7.00(/) |  | 1.50(0.99)  1.52(0.94) | 5.71(0.92)  5.30(0.54) |  |  |
| **Main Thoracic** | **Shanghai, *N(%)*** | 1808(95.0) | 63(3.3) | 26(1.4) | 6(0.3) | 3.23 | 1679(88.2) | 192(10.1) | 32(1.7) | 1.02 | 1808(95.0) | 95(5.0) | 0.14 |  |
|  | **Gansu, *N(%)*** | 1803(94.7) | 75(3.9) | 23(1.2) | 2(0.1) |  | 1678(88.2) | 200(10.5) | 25(1.3) |  | 1803(94.7) | 100(5.3) |  |  |
|  | **Shanghai**  ***VS* Gansu, *Mean(SD)*** | 1.82(1.07)  1.82(1.04) | 5.40(0.49)  5.45(0.50) | 7.27(0.60)  7.17(0.39) | 12.00(2.53)  10.0(0.00) |  | 1.65(0.92)  1.65(0.88) | 4.46(0.72)  4.55(0.77) | 8.16(2.20)  7.40(0.87) |  | 1.82(1.07)  1.82(1.04) | 6.33(1.87)  5.97(1.04) |  |  |
| **Lumbar** | **Shanghai, *N(%)*** | 1797(94.4) | 59(3.1) | 36(1.9) | 11(0.6) | 10.85* | 1649(86.7) | 207(10.9) | 47(2.5) | 2.89 | 1797(94.4) | 106(5.6) | 0.12 |  |
|  | **Gansu, *N(%)*** | 1792(94.2) | 77(4.0) | 33(1.7) | 1(0.1) |  | 1677(88.1) | 192(10.1) | 34(1.8) |  | 1792(94.2) | 111(5.8) |  |  |
|  | **Shanghai**  ***VS* Gansu, *Mean(SD)*** | 1.92(1.10)  1.78(1.03)** | 5.47(0.50) 5.48(0.50) | 7.64(0.76) 7.39(0.61) | 11.45(1.37) 11.00(/) |  | 1.73(0.95)  1.63(0.88)** | 4.42(0.72)  4.59(0.79)* | 8.53(1.88)  7.50(0.86)** |  | 1.92(1.10)  1.78(1.03)** | 6.83(2.00)  6.10(1.13)* |  |  |
| **Max** | **Shanghai, *N(%)*** | 1739(91.4) | 95(5.0) | 54(2.8) | 15(0.8) | 14.17** | 1480(77.8) | 354(18.6) | 69(3.6) | 3.29 | 1739(91.4) | 164(8.6) | 0.63 |  |
|  | **Gansu, *N(%)*** | 1725(90.6) | 125(6.6) | 51(2.7) | 2(0.1) |  | 1518(79.8) | 332(17.4) | 53(2.8) |  | 1725(90.6) | 178(9.4) |  |  |
|  | **Shanghai**  ***VS* Gansu, *Mean(SD)*** | 2.51(0.92)  2.33(0.95)** | 5.53(0.50) 5.52(0.50) | 7.46(0.69) 7.29(0.53) | 11.53(1.81) 10.50(0.71) |  | 2.25(0.73)  2.10(0.77)** | 4.41(0.73)  4.57(0.80)** | 8.35(1.98)  7.42(0.82)** |  | 2.51(0.92)  2.33(0.95)** | 6.71(1.93)  6.08(1.06)** |  |  |

**Note: Data are n (%) or mean (SD) unless specified otherwise. PSM, propensity score matching; ATR=the angle of trunk rotation. * *p*＜0.05, ** *p*＜0.01.**

**Reference**

1. GB/T 16133—2014. Screening of spinal curvature abnormality of children and adolescents[S]. In. People's Republic of China: National Health and Family Planning Commission of the People's Republic of China, China National Standardization Management Committee; 2015.

2. Adamczewska K, Wiernicka M, Malchrowicz-Mośko E, Małecka J, Lewandowski J. The Angle of Trunk Rotation in School Children: A Study from an Idiopathic Scoliosis Screening. Prevalence and Optimal Age Screening Value. *Int J Environ Res Public Health.* 2019;16(18):3426.

3. Negrini S, Donzelli S, Aulisa AG, et al. 2016 SOSORT guidelines: orthopaedic and rehabilitation treatment of idiopathic scoliosis during growth. *Scoliosis Spinal Disord.* 2018;13:3-3.
