# Supplementary material for: Regional disparity in epidemiological characteristics of adolescent scoliosis in China: Data from a screening program
Source: Front Public Health. 2022 Dec 6;10:935040. doi: 10.3389/fpubh.2022.935040 (PMC9764629; doi:10.3389/fpubh.2022.935040)
Supplement: Supplementary file 4 [file Table_4.docx]

**eTable4 Multivariate linear regression models in two regions.**

| **Region** | **Independent variable** | **Unstandardized coefficient** | | **Standardized coefficient** | **95% CI for β** | | **t** | **P value** | **Collinearity statistics** | |
| --- | --- | --- | --- | --- | --- | --- | --- | --- | --- | --- |
|  |  | **β** | **S. E** | **S. β** | **Lower** | **Upper** |  |  | **Tolerance** | **VIF** |
| ***Level1*** |  |  |  |  |  |  |  |  |  |  |
| **Shanghai** | **Gender(reference=Male)** | -0.040 | 0.033 | -0.020 | -0.105 | 0.025 | -1.216 | 0.224 | 0.906 | 1.103 |
|  | **Age(years)** | 0.017 | 0.017 | 0.019 | -0.015 | 0.050 | 1.043 | 0.297 | 0.711 | 1.407 |
|  | **BMI(kg/cm^2^)** | -0.008 | 0.004 | -0.032 | -0.016 | -0.000 | -2.011 | 0.044* | 0.908 | 1.101 |
| **Gansu** | **Gender(reference=Male)** | 0.242 | 0.045 | 0.116 | 0.154 | 0.330 | 5.398 | 0.000** | 0.851 | 1.176 |
|  | **Age(years)** | -0.089 | 0.023 | -0.081 | -0.134 | -0.043 | -3.815 | 0.000** | 0.867 | 1.153 |
|  | **BMI(kg/cm^2^)** | 0.012 | 0.007 | -0.037 | -0.025 | 0.001 | -1.842 | 0.066 | 0.992 | 1.009 |
| ***Level2*** |  |  |  |  |  |  |  |  |  |  |
| **Shanghai** | **Gender(reference=Male)** | -0.040 | 0.044 | -0.014 | -0.127 | 0.047 | -0.894 | 0.372 | 0.906 | 1.103 |
|  | **Age(years)** | 0.053 | 0.022 | 0.043 | 0.009 | 0.096 | 2.370 | 0.018* | 0.711 | 1.407 |
|  | **BMI(kg/cm^2^)** | -0.022 | 0.005 | -0.064 | -0.032 | -0.011 | -4.018 | 0.000** | 0.908 | 1.101 |
| **Gansu** | **Gender(reference=Male)** | 0.015 | 0.059 | 0.005 | -0.101 | 0.130 | 0.250 | 0.802 | 0.851 | 1.176 |
|  | **Age(years)** | -0.072 | 0.030 | -0.050 | -0.132 | -0.012 | -2.367 | 0.018* | 0.867 | 1.153 |
|  | **BMI(kg/cm^2^)** | -0.019 | 0.009 | -0.043 | -0.036 | -0.002 | -2.138 | 0.033* | 0.992 | 1.009 |
| ***Level3*** |  |  |  |  |  |  |  |  |  |  |
| **Shanghai** | **Gender(reference=Male)** | 0.048 | 0.048 | 0.016 | -0.047 | 0.143 | 0.995 | 0.320 | 0.906 | 1.103 |
|  | **Age(years)** | 0.040 | 0.024 | 0.030 | -0.008 | 0.087 | 1.634 | 0.102 | 0.711 | 1.407 |
|  | **BMI(kg/cm^2^)** | -0.038 | 0.006 | -0.104 | -0.050 | -0.027 | -6.474 | 0.000** | 0.908 | 1.101 |
| **Gansu** | **Gender(reference=Male)** | 0.116 | 0.062 | 0.040 | -0.005 | 0.237 | 1.874 | 0.061 | 0.851 | 1.176 |
|  | **Age(years)** | -0.103 | 0.032 | -0.069 | -0.165 | -0.040 | -3.213 | 0.001** | 0.867 | 1.153 |
|  | **BMI(kg/cm^2^)** | -0.015 | 0.009 | -0.033 | -0.033 | 0.003 | -1.656 | 0.098 | 0.992 | 1.009 |
| ***Max*** |  |  |  |  |  |  |  |  |  |  |
| **Shanghai** | **Gender(reference=Male)** | 0.057 | 0.046 | 0.020 | -0.033 | 0.147 | 1.237 | 0.216 | 0.906 | 1.103 |
|  | **Age(years)** | 0.066 | 0.023 | 0.052 | 0.021 | 0.111 | 2.869 | 0.004** | 0.711 | 1.407 |
|  | **BMI(kg/cm^2^)** | -0.039 | 0.006 | -0.110 | -0.049 | -0.028 | -6.881 | 0.000** | 0.908 | 1.101 |
| **Gansu** | **Gender(reference=Male)** | 0.137 | 0.062 | 0.048 | 0.016 | 0.258 | 2.218 | 0.027* | 0.851 | 1.176 |
|  | **Age(years)** | -0.096 | 0.032 | -0.064 | -0.158 | -0.033 | -3.000 | 0.003** | 0.867 | 1.153 |
|  | **BMI(kg/cm^2^)** | -0.031 | 0.009 | -0.068 | -0.049 | -0.013 | -3.411 | 0.001** | 0.992 | 1.009 |

**Note: BMI: Body Mass Index; VIF: Variance Inflation Factor; CI: Confidence Interval. * *p*＜0.05, ** *p*＜0.01.**
