# Supplementary material for: Regional disparity in epidemiological characteristics of adolescent scoliosis in China: Data from a screening program
Source: Front Public Health. 2022 Dec 6;10:935040. doi: 10.3389/fpubh.2022.935040 (PMC9764629; doi:10.3389/fpubh.2022.935040)
Supplement: Supplementary file 5 [file Table_5.docx]

**eTable 5.** **Multivariate linear regression models of age, region, and the interaction of age and region (PSM)**

| **Independent variable** | **Unstandardized coefficient** | | **Standardized coefficient** | **95% CI for β** | | **t** | **P value** | **Collinearity statistics** | |
| --- | --- | --- | --- | --- | --- | --- | --- | --- | --- |
|  | **β** | **S. E** | **S. β** | **Lower** | **Upper** |  |  | **Tolerance** | **VIF** |
| ***Model 1(Proximal Thoracic)*** | | | | | | | | | |
| **Region (reference=Shanghai)** | 0.079 | 0.036 | 0.038 | 0.009 | 0.15 | 2.211 | 0.027* | 0.893 | 1.119 |
| **Age_c** | -0.004 | 0.023 | -0.003 | -0.049 | 0.042 | -0.153 | 0.878 | 0.577 | 1.733 |
| **Region*Age_c** | -0.1 | 0.036 | -0.06 | -0.172 | -0.029 | -2.753 | 0.006** | 0.551 | 1.815 |
| ***Model 2(Main Thoracic)*** | | | | | | | | | |
| **Region (reference=Shanghai)** | 0.003 | 0.049 | 0.001 | -0.093 | 0.1 | 0.068 | 0.946 | 0.893 | 1.119 |
| **Age_c** | 0.034 | 0.032 | 0.023 | -0.028 | 0.097 | 1.073 | 0.283 | 0.577 | 1.733 |
| **Region*Age_c** | -0.063 | 0.05 | -0.027 | -0.161 | 0.035 | -1.253 | 0.210 | 0.551 | 1.815 |
| ***Model 3(Lumbar)*** | | | | | | | | | |
| **Region (reference=Shanghai)** | -0.124 | 0.053 | -0.04 | -0.228 | -0.021 | -2.367 | 0.018* | 0.893 | 1.119 |
| **Age_c** | 0.043 | 0.034 | 0.026 | -0.024 | 0.11 | 1.245 | 0.213 | 0.577 | 1.733 |
| **Region*Age_c** | -0.126 | 0.053 | -0.052 | -0.231 | -0.022 | -2.373 | 0.018* | 0.551 | 1.815 |
| ***Model 4(Max)*** | | | | | | | | | |
| **Region (reference=Shanghai)** | -0.153 | 0.052 | -0.051 | -0.255 | -0.052 | -2.966 | 0.003** | 0.893 | 1.119 |
| **Age_c** | 0.059 | 0.034 | 0.037 | -0.007 | 0.125 | 1.758 | 0.079 | 0.577 | 1.733 |
| **Region*Age_c** | -0.132 | 0.052 | -0.055 | -0.235 | -0.03 | -2.527 | 0.012* | 0.551 | 1.815 |

**Note: The models include body mass index and sex; those data were excluded from this table for simplicity. Age data were centralized to prevent collinearity after the interaction of the two variables. PSM, propensity score matching; CI, confidence interval; VIF, variance inflation factor.**
